# Altered lipid levels provide evidence for myelin dysfunction in multiple system atrophy

**DOI:** 10.1186/s40478-014-0150-6

**Published:** 2014-10-29

**Authors:** Anthony S Don, Jen-Hsiang T Hsiao, Jonathan M Bleasel, Timothy A Couttas, Glenda M Halliday, Woojin Scott Kim

**Affiliations:** Prince of Wales Clinical School, Faculty of Medicine, University of New South Wales, Sydney, NSW 2052 Australia; Neuroscience Research Australia, Barker St, Randwick, NSW 2031 Australia; School of Medical Sciences, University of New South Wales, Sydney, NSW 2052 Australia

**Keywords:** Multiple system atrophy, Myelin, Lipid, α-synuclein, Sphingomyelin, Sulfatide, Galactosylceramide, MSA white matter

## Abstract

Multiple system atrophy (MSA) is a rapidly-progressive neurodegenerative disease characterized by parkinsonism, cerebellar ataxia and autonomic failure. A pathological hallmark of MSA is the presence of α-synuclein deposits in oligodendrocytes, the myelin-producing support cells of the brain. Brain pathology and *in vitro* studies indicate that myelin instability may be an early event in the pathogenesis of MSA. Lipid is a major constituent (78% w/w) of myelin and has been implicated in myelin dysfunction in MSA. However, changes, if any, in lipid level/distribution in MSA brain are unknown. Here, we undertook a comprehensive analysis of MSA myelin. We quantitatively measured three groups of lipids, sphingomyelin, sulfatide and galactosylceramide, which are all important in myelin integrity and function, in affected (under the motor cortex) and unaffected (under the visual cortex) white matter regions. For all three groups of lipids, most of the species were severely decreased (40–69%) in affected but not unaffected MSA white matter. An analysis of the distribution of lipid species showed no significant shift in fatty acid chain length/content with MSA. The decrease in lipid levels was concomitant with increased α-synuclein expression. These data indicate that the absolute levels, and not distribution, of myelin lipids are altered in MSA, and provide evidence for myelin lipid dysfunction in MSA pathology. We propose that dysregulation of myelin lipids in the course of MSA pathogenesis may trigger myelin instability.

## Introduction

Multiple system atrophy (MSA) is a fatal neurodegenerative disease characterized by parkinsonism, cerebellar ataxia and autonomic failure. The disease affects older individuals with an incidence in the 50–99 year age group of 3 per 100,000 [1,2]. The mean survival after diagnosis is 9 years [3,4]. MSA is defined histopathologically by the accumulation of α-synuclein protein mainly in the cytoplasm of oligodendrocytes, the myelin producing support cells of the central nervous system [5]. These glial cytoplasmic inclusions (GCIs) concentrate in the striatonigral system, olivopontocerebellar system and autonomic nuclei of the brain stem and spinal chord [6,7]. Neurodegeneration in these areas is thought to follow loss of trophic and metabolic support provided by ensheathing oligodendrocytes [8–10].

The pathology of MSA contrasts with Parkinson’s disease (PD) and dementia with Lewy bodies (DLB) where α-synuclein accumulation and toxicity occurs in neurons. The reason for this fundamental difference in cellular focus remains poorly understood. In further contrast to PD/DLB, no pathogenic mutations or risk-conferring loci on the α-synuclein gene, *SNCA*, have been associated with MSA [11–14]. Thus there is a need to identify novel oligodendrocyte-specific pathways underlying the early pathogenesis of this disease. In this respect, recent progress in the study of oligodendrocyte lipid handling and myelin maintenance heralds a promising advance.

Disruption of myelin structural proteins has been observed preceding visible GCIs in postmortem samples of MSA patients [15], suggesting that myelin instability may be an early event in the pathogenesis of MSA. Myelin stability in turn is dependent on the content and inter-association of its lipid constituents, particularly cholesterol and the ceramide-derived sphingolipids sphingomyelin, sulfatide and galactosylceramide [16–19]. Lipid homeostasis is increasingly recognized as a crucial factor for normal brain function and growing evidence shows that altered lipid levels and/or distribution are contributory factors in a number of neurodegenerative diseases. Recently, the lipid transporter ABCA8 was shown to stimulate sphingomyelin production in oligodendrocytes and likely to have a role in myelin processing and maintenance [20]. These data provide strong impetus for investigation of myelin lipid dysregulation in MSA pathology.

**Figure 1:**
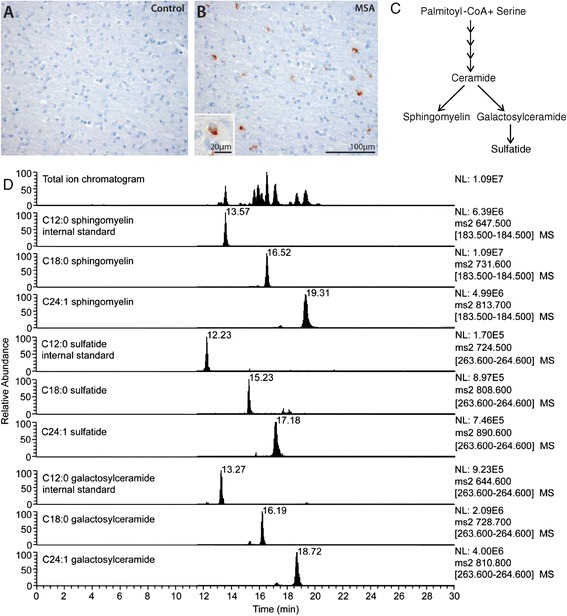
**Analysis of MSA brain tissues.** Analysis of cells using Nissl staining and GCIs using α-synuclein immunohistochemistry in the white matter underlying the motor cortex of MSA and control brain **(A,B)**. Despite obvious α-synuclein accumulation in GCIs only in MSA, no significant cell loss or other tissue alterations were detected. The biosynthetic pathway of myelin lipids sphingomyelin, sulfatide and galactosylceramide **(C)**. Extracted ion chromatograms for sphingomyelin, sulfatide and galactosylceramide species **(D)**. Total and extracted ion chromatograms are shown for C12:0 internal standards, as well as native C18:0 and C24:1 variants for each lipid class, in a control MC white matter tissue sample. Individual MS scans are shown rather than smoothed peaks. Each individual ion peak was comprised of a minimum of 10 MS scans.

Although lipid constitutes 78% of myelin [21] and myelin dysregulation is recognized as an important early pathological event in MSA [15], changes in lipid level/distribution in MSA brain is unknown. In this study we assess the quantitative changes in sphingomyelin and the important structural myelin lipids sulfatide and galactosylceramide in a MSA-affected and unaffected brain region. Galactosylceramide is formed in oligodendrocytes through the action of ceramide galactosyltransferase (CGT) [22,23]. Galactosylceramide may then be further modified to sulfatide through the action of a specific sulfotransferase (Figure 1). Thus we chose to investigate these lipids as an indicator of oligodendrocyte-specific myelin lipid metabolism. We hypothesize that regulation of these lipid species is altered in MSA pathogenesis, inducing myelin instability and disruption of proteins associated with the lipid membrane.

## Materials and methods

### Human brain tissues

Human brain tissues were obtained from the Sydney Brain Bank and NSW Tissue Resource Centre, part of the Australian Brain Bank Network funded by the National Health and Medical Research Council of Australia. Ethics approval was from the University of New South Wales Human Research Ethics Committee. Frozen brain tissues from 8 MSA cases and 10 controls were used in this study. MSA brains were clinically and pathologically diagnosed using international diagnostic criteria [24], and were free of Lewy bodies. Controls were free of significant neuropathology. Clinical information for each case including gender, age at death, postmortem interval (PMI), and disease duration is provided in Table 1. The mean age of control and MSA subjects was 77.4 and 67.8 respectively (p = 0.02), which is a potential limitation of the present study cohort. The mean PMI was 21.4 and 16.9 respectively (p = 0.45). Approximately 50 mg of brain tissue from anatomically specified regions were collected using a 3-mm stainless steel biopsy needle from frozen brain slices (dissected on a bed of dry-ice).

**Table 1:** **Demographics of MSA and control cases**

**Brain ID**	**Age**	**Gender**	**PMI (h)**	**MSA-P/MSA-C**	**Disease duration (y)**
**MSA**					
MSA1	61	male	21	MSA-P	4
MSA2	82	male	8	MSA-P	7
MSA3	64	male	22	MSA-P	8
MSA4	61	male	7	MSA-P	2
MSA5	62	male	31	MSA-C	10
MSA6	71	female	19	MSA-P	6
MSA7	74	male	16	MSA-P	10
MSA8	67	male	11	MSA-P	8
**Control**					
Con1	78	female	37	N/A	N/A
Con2	82	female	7.5	N/A	N/A
Con3	69	male	13.5	N/A	N/A
Con4	85	male	9	N/A	N/A
Con5	65	male	14.5	N/A	N/A
Con6	68	male	45.5	N/A	N/A
Con7	73	male	38.5	N/A	N/A
Con8	88	male	9	N/A	N/A
Con9	85	female	10	N/A	N/A
Con10	81	male	29	N/A	N/A

### Detection of GCIs

Formalin-fixed coronal blocks of white matter and overlying motor cortex were paraffin-embedded, cut at 10 μm on a microtome, and mounted on 3-aminopropyltriethoxysilane-coated slides. Following pretreatment with 99% formic acid for 3 min, immunoperoxidase staining was performed using antibodies to α-synuclein (mouse mAb42, BD Transduction Labs, USA; diluted 1:100) and an avidin-biotin-peroxidase detection system (Vector Laboratories, Burlingame, CA, USA). Sections were counterstained with 0.5% cresyl violet to identify cell constituents. Labeled sections were evaluated and photographed using an Olympus BX51 fluorescence microscope fitted with specific filter systems and a computerized image analysis system (SPOT camera, Image Pro Plus software).

### Lipid assay

Lipids were quantified using Liquid Chromatography-tandem Mass Spectrometry (LC-MS/MS). Brain tissue samples were accurately weighed, i.e. 20 mg wet weight, and lipids were extracted from brain tissues according to a previously published one-phase extraction protocol [25]. Lipids were resolved and quantified by LC-MS/MS, as described previously [26], with the exception that 1250 rather than 250 pmoles C12:0 sphingomyelin (Avanti Polar Lipids, AL, USA) internal standard was added to each sample. C12:0 sulfatide and C12:0 galactosylceramide internal standards were added at 250 pmoles/sample. Data processing was carried out using the MMSAT program [26] and the detected sphingomyelin and sulfatide species were verified as conforming to a previously identified quadratic elution profile [27]. Lipids, expressed as ratios to the relevant internal standard, were quantified using standard curves prepared with sphingomyelin, galactosylceramide, and sulfatide external standards (Avanti Polar Lipids).

### RNA isolation, reverse transcription and quantitative PCR

RNA was isolated using TRI Reagent (Sigma, Castle Hill, NSW, Australia) following the manufacturer’s protocol from control (n = 10) and MSA (n = 8) tissues. All procedures were carried out using RNase-free reagents and consumables. Five micrograms of RNA was reverse transcribed into cDNA using Moloney-murine leukemia virus reverse transcriptase and random primers (Promega, Annandale, NSW, Australia) in a 20 μl reaction volume. cDNA was used as a template in the quantitative real-time PCR (qPCR) assay, which was carried out using a Mastercycler ep realplex S (Eppendorf) and the fluorescent dye SYBR Green (Bio-Rad), following the manufacturer’s protocol. Briefly, each reaction (20 μl) contained 1x RealMasterMix, 1x SYBR green, 5 pmoles of primers and 1 μl of template. Amplification was carried out with 40 cycles of 94°C for 15 sec and 60°C for 1 min. Gene expression was normalized to the housekeeper gene β-actin. The primers used were: α-synuclein F:TAGGCTCCAAAACCAAGGAGG R:CCTTCTTCATTCTTGCCCAACT; β-actin F:TCATGAAGTGTGACGTGGACATCCGT R:CCTAGAAGCATTTGCGGTGCACGATG.

The level of expression was calculated using the comparative threshold cycle (Ct) value method using the formula 2^−∆∆Ct^ (where ∆∆Ct = ∆Ct sample – ∆Ct reference).

### Protein extraction

To 50 mg tissue, 250 μL of ice-cold hypotonic buffer (250 mM sucrose, 10 mM HEPES (pH 7.4), 1 mM EDTA) containing complete protease inhibitors (1 mM benzamidine, 1 μg/mL leupeptin, 1 μg/mL aprotinin, 1 μg/mL pepstatin-A; Roche Applied Science, Castle Hill, NSW, Australia) was added. Tissues were dounced 20 times in microfuge tubes using tight pestles (Sigma, Castle Hill, NSW, Australia) while on ice. The tubes were centrifuged at 800 *g* for 10 min at 4°C and the supernatants were transferred to new tubes and stored at −20°C.

### Western blotting

Protein concentrations were determined using BCA protein assay. Equal amounts of protein from control (n = 10) and MSA (n = 8) samples were separated on SDS-PAGE gels (12% acrylamide) and transferred onto 0.45 μm nitrocellulose membranes at 100 volts for 30 min. Membranes were blocked overnight at 4°C in PBS containing 5% non-fat dry milk and probed with primary antibody (1/1000 dilution) at 4°C overnight. The membranes were washed three times in PBS containing 0.1% Tween-20 and then incubated with horseradish peroxidase-conjugated secondary antibody (Dako, Carpinteria, CA, USA, 1/2000 dilution) for 2 h. Signals were detected using enhanced chemiluminescence (ECL, GE Healthcare, Buckinghamshire, UK) and X-ray films. The signal intensity was quantified using NIH ImageJ software.

### Statistical analysis

MSA and control human tissue samples examined were n = 8 and n = 10 respectively. Data presented are expressed as mean ± SE shown by the error bars; statistical outliers, identified using the ROUT method with GraphPad PRISM software (Q =1%), were excluded from the analysis. Statistical significance was analyzed using the Student’s *t* test with a *p* <0.05 considered significant.

## Results

### Analysis of MSA brain tissues

To determine if altered white matter lipid levels are a contributory factor in MSA pathogenesis we undertook a case–control analysis of lipids in an affected and unaffected white matter region. The affected region was the white matter underlying the primary motor cortex (MC) from the most superior and medial aspect of the coronal slice. This region in MSA has GCIs but no significant cell loss or alteration in tissue composition (Figure 1A,B), whereas the white matter underlying the visual cortex (VC) is unaffected by MSA pathology and is commonly used as a disease-unaffected control region [28]. We extracted white matter lipids from freshly-frozen brain and analyzed sphingomyelin, sulfatide and galactosylceramide, three of the major lipids in myelin (Figure 1C), using LC-MS/MS. The chromatograms for major sphingomyelin, sulfatide and galactosylceramide species are shown to indicate lipid measurement quality (Figure 1D).

### Decreased sphingomyelin levels in MSA white matter

**Figure 2:**
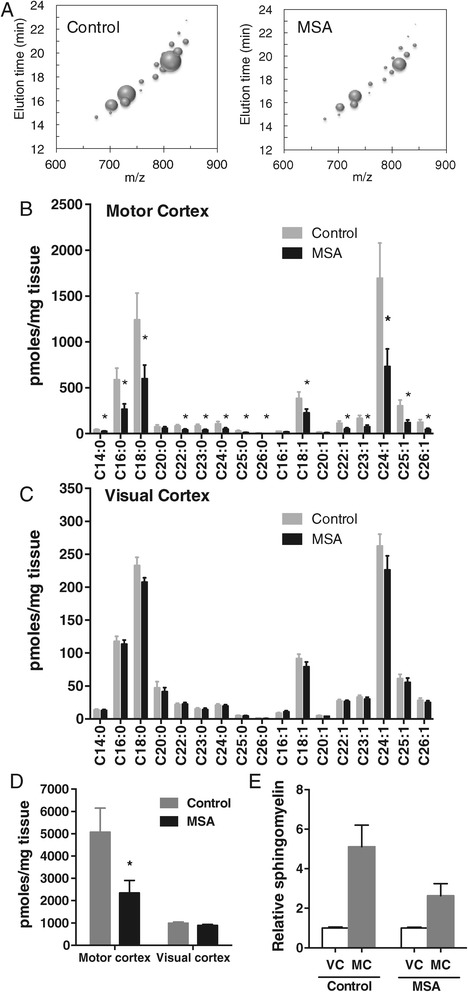
**Analysis of sphingomyelin levels in MSA and control white matter. (A)** Bubble plots illustrating the mass/charge ratio (*m/z*), column elution time, and relative abundance (size of the bubbles) of sphingomyelin species detected in the MC white matter. **(B,C)** Bar graphs showing the absolute amounts of different sphingomyelin species in the MSA-affected (MC) and unaffected (VC) white matter. **(D)** A comparison of the total sphingomyelin levels. **(E)** The relative sphingomyelin levels in VC and MC. Data represent mean and SE as error bars, *p < 0.05.

A bubble plot shows the decrease in volume size (indicative of lipid amount) of individual sphingomyelin species in the MSA-affected white matter (Figure 2A). The level of almost all sphingomyelin species was selectively decreased (or showed a trend for decrease) only in the MSA-affected white matter compared to controls (Figure 2B,C); the decrease was substantial, i.e. 40 – 63%. A comparison of the total sphingomyelin level (sum of all sphingomyelin species) clearly showed a significant and selective difference between the two groups only in the MSA-affected white matter (Figure 2D). The relative sphingomyelin levels in VC and MC white matter were also assessed. In controls, the level of sphingomyelin was 4.3 fold higher in MC compared to VC white matter, whereas in MSA the level of sphingomyelin was only 2.0-fold higher in MC compared to VC white matter (Figure 2E), indicating, once again, a selective decrease in sphingomyelin levels in the MSA-affected white matter.

**Figure 3:**
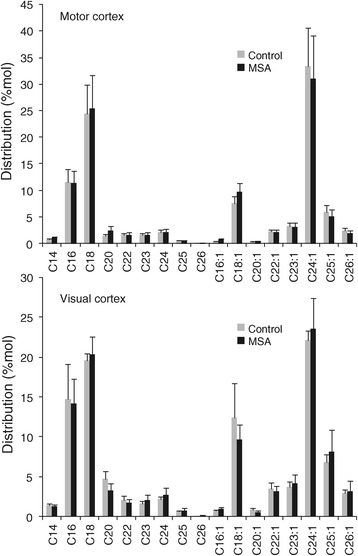
**Distribution of different sphingomyelin species in the MSA-affected (MC) and unaffected (VC) white matter.**

To evaluate whether the distribution of different sphingomyelin species changed with MSA, the level of each sphingomyelin species was calculated as a percentage of total sphingomyelin level in the two white matter regions. Overall, the distribution of sphingomyelin species was similar in the two groups in both white matter regions (Figure 3). No significant shift in white matter fatty acid chain length/content was evident with MSA. It was interesting to note that the distribution of white matter sphingomyelin species was overall similar in the two regions evaluated. These data show that the absolute levels, and not distribution, of white matter sphingomyelin species are altered by MSA.

### Decreased sulfatide levels in MSA white matter

**Figure 4:**
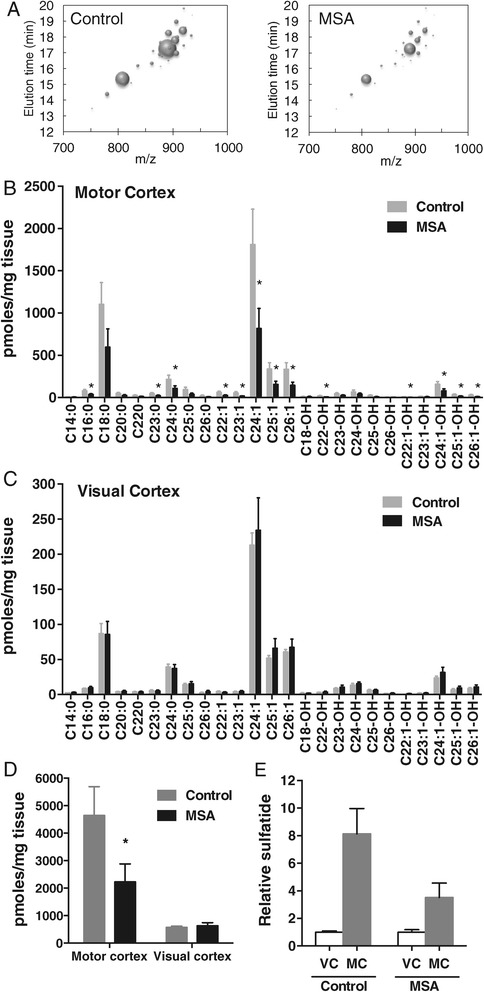
**Analysis of sulfatide levels in MSA and control white matter. (A)** Bubble plots illustrating the mass/charge ratio (*m/z*), column elution time, and relative abundance (size of the bubbles) of sulfatide species detected in the MC white matter. **(B,C)** Bar graphs showing the absolute amounts of different sulfatide species in the MSA-affected (MC) and unaffected (VC) white matter. **(D)** A comparison of the total sulfatide levels. **(E)** The relative sulfatide levels in VC and MC. Data represent mean and SE as error bars, *p < 0.05.

Similarly, we analyzed sulfatide levels in the same white matter tissue samples; sulfatide is thought to play a major role in myelin function and stability, which is important in MSA pathogenesis. A bubble plot shows the decrease in volume size of individual sulfatide species in MSA-affected white matter (Figure 4A). In MSA-affected white matter, the level of most sulfatide species was significantly decreased, i.e. 48 – 68% (Figure 4B), whereas in unaffected white matter, no significant difference was detected (Figure 4C). As expected, a comparison of the total sulfatide levels (sum of all sulfatide species) showed a significant difference between the two groups only in MSA-affected white matter (Figure 4D). The relative levels of sulfatide in the two white matter regions were also assessed. In controls, the level of sulfatide was 4.0-fold higher in MC compared to VC white matter, whereas in MSA the level of sulfatide was only 2.7-fold higher in MC compared to VC white matter (Figure 4E).

**Figure 5:**
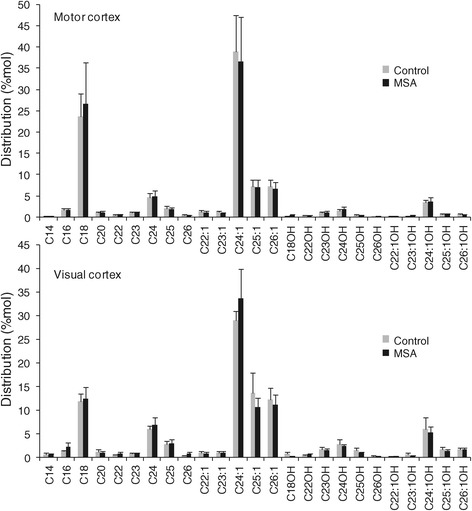
**Distribution of different sulfatide species in the MSA-affected (MC) and unaffected (VC) white matter.**

To evaluate whether the distribution of different white matter sulfatide species changed with MSA, each sulfatide species was calculated as a percentage of total sulfatide level in the two white matter regions (Figure 5). Once again, there was no significant difference in the distribution of each sulfatide species in either of white matter region; no shift in fatty acid chain length/content of white matter sulfatide with MSA. These data clearly show that the absolute levels, and not distribution, of white matter sulfatide species are altered in MSA.

### Decreased galactosylceramide levels in MSA white matter

**Figure 6:**
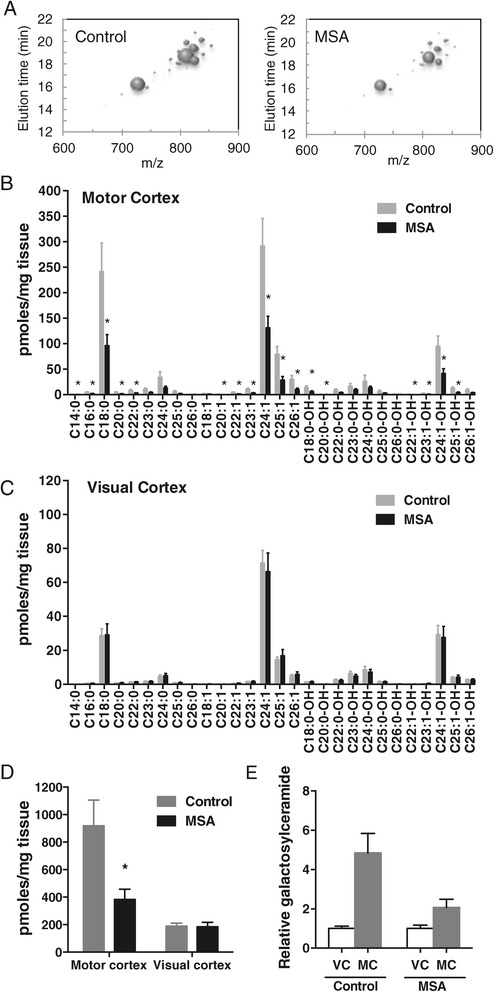
**Analysis of galactosylceramide levels in MSA and control white matter. (A)** Bubble plots illustrating the mass/charge ratio (*m/z*), column elution time, and relative abundance (size of the bubbles) of galactosylceramide species detected in the MC white matter. **(B,C)** Bar graphs showing the absolute amounts of different galactosylceramide species in the MSA-affected (MC) and unaffected (VC) white matter. **(D)** A comparison of the total galactosylceramide levels. **(E)** The relative galactosylceramide levels in VC and MC. Data represent mean and SE as error bars, *p < 0.05.

**Figure 7:**
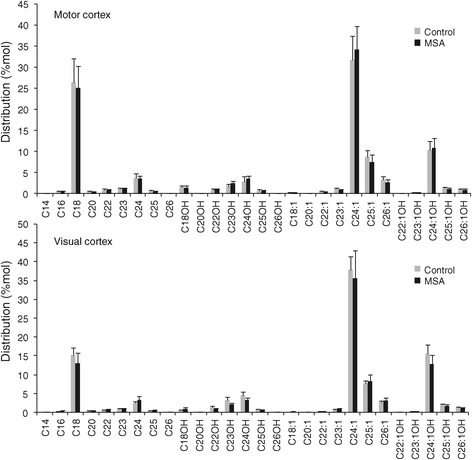
**Distribution of different galactosylceramide species in the MSA-affected (MC) and unaffected (VC) white matter.**

**Figure 8:**
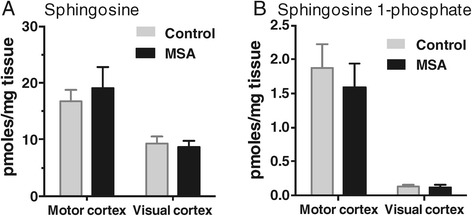
**Analysis of sphingosine and sphingosine 1-phosphate levels in MSA and control white matter.** Bar graphs showing the absolute amounts of sphingosine **(A)** and sphingosine 1-phosphate **(B)** in the MSA-affected (motor cortex) and unaffected (visual cortex) white matter. Data represent mean and SE as error bars.

Again, we analyzed galactosylceramide levels in the same white matter tissue samples; galactosylceramide is a myelin structural lipid and biosynthetic precursor to sulfatide. Decreased abundance of galactosylceramide species in MC white matter of MSA cases is illustrated in a bubble plot (Figure 6A). In MSA-affected white matter, the level of most galactosylceramide species was significantly decreased, i.e. 55 – 69% (Figure 6B), whereas in unaffected white matter, no significant difference was detected (Figure 6C). A comparison of the total white matter galactosylceramide levels (sum of all galactosylceramide species) showed a significant difference in the two groups only in MSA-affected white matter (Figure 6D). The level of galactosylceramide was 4.9-fold higher in MC compared to VC white matter in controls, and only 1.7-fold higher in MSA (Figure 6E). Once again, there was no significant difference in the distribution of each galactosylceramide species in either of the white matter regions (Figure 7). In addition, we have analyzed two more lipids, sphingosine and sphingosine 1-phosphate, to determine whether changes to lipid levels are universal or specific to certain lipids. We found that neither sphingosine nor sphingosine 1-phosphate levels changed in the MC white matter of MSA (Figure 8), suggesting that not all lipid metabolism is altered.

### Analysis of α-synuclein expression in MSA white matter

**Figure 9:**
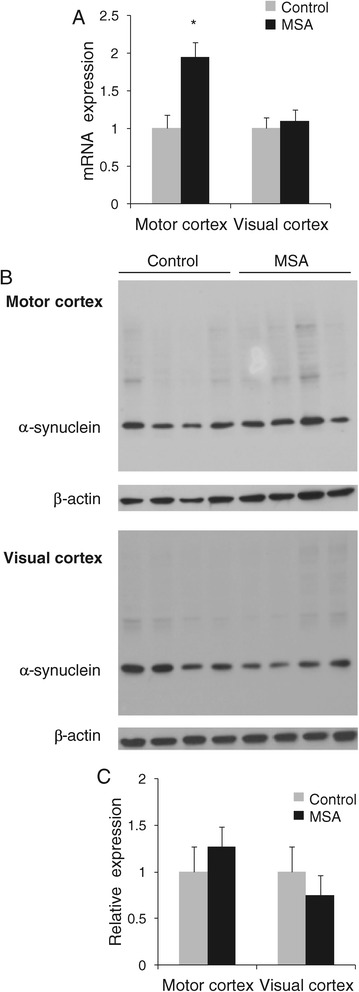
**Analysis of α-synuclein expression in MSA (n = 8) and control (n = 10) white matter. (A)** mRNA expression of α-synuclein as measured by qPCR; data represent mean and SE as error bars, *p < 0.05. **(B)** Soluble cytosolic α-synuclein protein as measured by western blotting of Control 1–4 and MSA 1–4. β-actin was used as a loading control. **(C)** Relative optical density of α-synuclein-immunoreactive protein bands as quantified using ImageJ software.

Since lipid levels were selectively decreased in the MSA-affected white matter and given the fact that α-synuclein aggregation process is affected by lipid level/composition, we were interested if α-synuclein expression is altered in the MSA-affected white matter. Aggregated α-synuclein is a major component of GCIs in MSA oligodendrocytes (Figure 1A) and is thought to play a pivotal role in MSA pathogenesis. Using the same tissue samples that were used in the lipid analysis, we isolated mRNA and soluble cytosolic protein and measured α-synuclein expression by qPCR and western blotting. The mRNA expression of α-synuclein was significantly elevated only in the MSA-affected white matter and was unaltered in the unaffected white matter (Figure 9A). The level of soluble cytosolic α-synuclein protein was not significantly altered in the MSA tissues (Figure 9B,C).

## Discussion

This quantitative case–control analysis provides novel insight into the altered myelin lipid milieu found in MSA-affected white matter. Total sphingomyelin levels were selectively decreased in the disease-affected white matter. Sphingomyelin is derived from ceramide through the action of sphingomyelin synthases. It is the most abundant sphingolipid in biological membranes and thus has a functional significance in almost all tissues. The myelin membrane of oligodendrocytes is particularly enriched with sphingomyelin. Since myelin comprises the bulk of lipid content in both grey and white matter regions – though to a much greater extent in the latter – total sphingomyelin content of whole brain homogenates is most likely a reflection of oligodendrocyte lipid metabolism.

Galactosylceramide and sulfatide levels were also selectively reduced in MSA-affected white matter. Ceramide galactosyltransferase (CGT) converts ceramide to galactosylceramide, which can be further modified to sulfatide through the action of a sulfotransferase. In contrast to sphingomyelin synthases, CGT has a restricted expression pattern with high concentration in oligodendrocytes, Schwann cells, kidney, testes and intestine [29]. Although there are no lipid species exclusively present in myelin, galactosylceramide and sulfatide are the most typical myelin lipids with concentrations in white matter approximately 10-fold higher than grey matter [30]. Thus the reduction of total galactosylceramide and sulfatide in MSA-affected white matter may suggest that oligodendrocyte handling of these myelin lipids is disrupted in the disease process along with sphingomyelin. Whilst our results clearly indicate a loss of myelin lipids in MSA-affected white matter, we cannot rule out the possibility that neuronal membrane lipids that are not present in myelin, such as certain gangliosides, also decline significantly, as these lipids were not assayed in the current study. However, there was no reduction in levels of the signaling lipids sphingosine and sphingosine 1-phosphate in MC white matter of MSA brains. These lipids represent a different branch of basic sphingolipid metabolism that is apparently unaffected in MSA, indicating that MSA does not adversely impact on all aspects of lipid metabolism.

Sphingomyelin and sulfatide species in mammalian cells display high variability in the N-acyl fatty acid chain, which ranges in length from C14 to C26 in all organs except skin, where the N-acyl chain is often longer. This variability is generated during the *de novo* synthesis of the common precursor molecule ceramide, which is regulated by six different ceramide synthase enzymes (CerS), each the product of a unique gene (reviewed in [31]). CerS catalyze the N-acylation of sphinganine or sphingosine with one of several possible fatty acyl-CoAs to produce dihydroceramide and ceramide respectively. The six CerS have distinct but overlapping acyl-chain preference, characterization of which has advanced understanding of the regulation of ceramide synthesis [32–37]. However, the biological significance of the length of fatty acid found in different ceramide and sphingolipid species remains obscure. It has been proposed that the fatty acid composition of ceramide and its derivatives could influence the biophysical properties of the membrane lipid bilayer or the activation of different lipid signaling pathways [38,39]. Due to the prominence of ceramide derivatives in the CNS lipid milieu, there is increasing interest in the role of fatty acid chain variants in neurological physiology and disease (see review [40]). Nonetheless, due to the relative nascence of this field of study, interpretation of our results concerning the differential expression of sphingomyelin and sulfatide subspecies in MSA compared with control brain samples is necessarily speculative.

With increasing age levels of very long chain C24 fatty acids in sphingomyelin increase, while those of C18 acids diminish [40]. Myelin galactosylceramide and sulfatide are also enriched with very-long chain fatty acids (C22-C24) [30]. Becker and colleagues [41] studied the cell and age-specific expression of CerS by means of Northern blot, quantitative PCR and *in situ* hybridization. Cer2, which uses very long chain acyl CoAs (C22-24) for ceramide synthesis, was abundantly expressed in all white matter tracts of the mouse brain and *in situ* hybridization confirmed strong expression in oligodendrocytes. In addition the phenotype of a CerS2 knockout mouse featured progressive myelin disorganization including failure of compaction and loss of myelin basic protein (MBP) [42]. Thus sphingolipids with long chain fatty acids, derived from CerS2 acylated precursors, appear to be essential for myelin production and maintenance.

In the present study we can observe that C24-sphingomyelin predominates in oligodendrocyte-rich white matter and the C24 variant of the myelin-typical lipid sulfatide is also abundant in white matter. This corroborates the previously reported enrichment of very-long chain fatty acids in myelin sphingolipids. However, the reductions in particular sphingolipid species observed in MSA-affected white matter compared to controls do not appear to be limited to a specific acyl-chain variant of sphingomyelin or sulfatide. This contrasts with the selective findings [40] of specific sphingolipid variant derangement in neurodegenerative conditions. The present results may point to a more general secondary dysregulation of myelin membrane components in MSA pathogenesis rather than a specific derangement of oligodendrocyte sphingolipid synthesis. Studies of CerS activities and other regulatory factors in sphingolipid synthesis are needed to further characterize the role of specific fatty acid chain variants in MSA and other conditions.

Song and colleagues observed abnormal relocalization and breakdown of myelin-associated proteins, p25α and MBP, which appeared to precede visible GCIs formation in affected oligodendrocytes [15]. The pathogenic protein α-synuclein is a major component of GCIs in MSA oligodendrocytes and we show that its expression at the transcriptional level was upregulated selectively in the MSA-affected white matter. The soluble cytosolic α-synuclein level was unaltered in the MSA tissues, indicating that α-synuclein present in MSA oligodendrocytes is likely to be in insoluble aggregated form, as a component of GCIs, which we clearly observed in the MSA-affected white matter (Figure 1A,B). The solubility of α-synuclein in MSA is quite different from that of PD or DLB. In MSA brain, α-synuclein is present mainly as SDS-soluble (not insoluble) accumulation. Detergent-insoluble α-synuclein is usually not detected [43]. This is in contrast to PD and DLB, in which α-synuclein is clearly detected in urea extracts of SDS-insoluble brain tissues [43,44]. The cross-sectional observations of the present case–control study cannot be used to make explicit temporal inferences regarding lipid dysregulation in MSA pathogenesis. Nonetheless depletion of sulfatide and sphingomyelin would fit well as precursors to myelin protein disruption in the working model of disease events.

The origin of α-synuclein in the oligodendrocyte GCIs remains enigmatic. Although the evidence of significant physiological expression of α-synuclein in mature oligodendrocytes is conflicting [45–47], it has been proposed that upregulation of the SNCA gene in these cells could be the cause of GCI formation. However, there appears to be no dysregulation of α-synuclein expression in the MSA disease state. In contrast, successful animal models of MSA, which recapitulate neuropathological features, have been generated by α-synuclein overexpression in the oligodendrocytes of mouse brains [48–50]. Alternatively, aberrant uptake of α-synuclein protein from the extracellular environment has also been proposed as a possible mechanism of GCI formation [45,51,52].

Separate lines of evidence from transgenic mouse models support the specific functional importance of myelin sulfatide content in the CNS. CGT knockout mice lacking both galactosylceramide and sulfatide have a neuropathological phenotype with tremor and severe motor weakness [22,23]. However, these deficits are abolished by targeted restoration of CGT function to oligodendrocytes only. Myelin composition and stability is also disturbed in mice lacking sulfatide with normal galactosylceramide levels, showing relatively normal early development of oligodendrocytes with later defects in myelin compaction and maintenance of protein domains in nodal regions [16].

Depletion of sphingomyelin in the MSA disease process is consistent with the observation of transcriptional upregulation of the lipid transporter ABCA8 in MSA-affected white matter. Previously, a region and age-specific study of ABCA8 expression in the human brain [20] showed selective enrichment in white matter regions and upregulation across the human life-span correlating with age-associated expansion of myelin [53,54]. *In vitro* ABCA8 was able to significantly stimulate both sphingomyelin synthase 1 expression and sphingomyelin production in a human oligodendrocyte cell line [20]. Thus ABCA8 is likely to be involved in oligodendrocyte sphingolipid metabolism and myelin synthesis and maintenance. In conjunction with the findings of the present study, ABCA8 upregulation may be a compensatory response to disturbance of sphingolipid metabolism.
